# Early intervention in cerebral palsy 1.0–3.0: From passive approaches to precision, context-responsive care

**DOI:** 10.1177/00368504261478184

**Published:** 2026-08-10

**Authors:** Iona Novak, Jens Bo Nielsen, Rachel Byrne, Christina Hoei-Hansen, Jessica Gengaroli, Karin Lind, Jenna Mitchell, Maria Mc Namara, Anina Ritterband-Rosenbaum, Catherine Morgan

**Affiliations:** 1 Cerebral Palsy Alliance Research Institute, Specialty of Child & Adolescent Health, Sydney Medical School, Faculty of Medicine & Health, 4334 The University of Sydney, NSW, Australia; 2 Department of Neuroscience, 4321 University of Copenhagen, Copenhagen, Denmark; 3 459814 Cerebral Palsy Foundation, New York, NY, USA; 4 Department of Paediatrics, Marys Center for Cerebral Palsy, University Hospital Rigshospitalet, Copenhagen, Denmark; 5 Department of Clinical Medicine, 4321 University of Copenhagen, Denmark; 6 503792 The Elsass Foundation, Charlottenlund, Denmark

**Keywords:** cerebral palsy, early intervention, neuroplasticity, rehabilitation, precision medicine

## Abstract

Cerebral palsy (CP) is the most common lifelong physical disability, with substantial global health and socioeconomic burden. Advances in early diagnosis now enable identification in infancy, enabling a critical window for intervention during periods of heightened neuroplasticity. This narrative review synthesises the evolution of early intervention from historical passive, late, and low-dose approaches to contemporary models emphasising early, child-active, task-specific, and contextually embedded care. Early randomised trials demonstrated benefits of active interventions compared with passive care. In contrast, more recent large pragmatic trials show attenuated overall between-group differences, with effects emerging primarily in subgroups defined by age, severity, and context. Evidence certainty is low to moderate, with the most consistent improvements observed in upper limb function in unilateral CP. Collectively, findings indicate that early intervention is effective, but not uniformly so, and that outcomes depend on the interaction between the individual, intervention, and environment. Early diagnostic and intervention pathways also provide earlier certainty regarding neurodevelopmental trajectory and enable timely identification of infants with treatable genetic conditions. The field is transitioning toward a precision medicine early intervention model focused on optimising who, when, how much, and where to intervene, reflecting a shift toward a precision early intervention model.

## 1. Introduction

### 1.1. Why early intervention matters

Cerebral palsy (CP) is the most common lifelong physical disability affecting posture and movement^1^ and represents a major global public health challenge, impacting an estimated 50 million people worldwide. Although prevalence has declined by approximately 40% in high-income countries,^2^ reflecting advances in antenatal and perinatal care within the first 1,000 days, it remains almost three times higher in low- and middle-income settings,^3^ underscoring persistent inequities in early detection, prevention, and intervention. The economic burden of CP is substantial at a global level. With lifetime costs of approximately $1.6 million per person in high-income settings, the total global economic burden is estimated to be in the order of tens of trillions of US dollars.^4^

The clinical and economic burden is profound and is borne by families and the health system. CP has no cure, with decline in mobility over time and features of accelerated ageing.^5^ One-third of individuals will never walk independently and half have cognitive impairments, limiting independence and participation.^5^ Multimorbidity is the norm rather than the exception, with high rates of chronic pain, communication impairment, epilepsy, behavioural disorders, sleep disturbance, and visual impairment.^5^ Those most affected experience poorer health, greater health inequities, and higher unemployment (29% vs 82%).^5^ Despite this burden, no single intervention alters severity at a population level, and the marked heterogeneity of CP necessitates lifelong, individualised, and multidisciplinary care.

A paradigm shift has occurred. Since the 2017 international guidelines,^6^ CP can be diagnosed accurately in infancy, enabling intervention during critical periods of neuroplasticity. Early diagnosis is rapidly becoming standard of care, creating a window of opportunity to alter developmental trajectories rather than merely compensate for established disability.

### 1.2. What is not yet clear

This shift has exposed a critical gap between early detection and optimal intervention. Clinicians must now determine not simply whether to intervene, but which interventions to use, for whom, and at what dose and timing. Many allied health professionals have limited experience working with infants with CP, reflecting a historical model in which diagnosis, and therefore intervention, typically occurred later in childhood. Although the evidence base has expanded rapidly, this growth has outpaced workforce capability, resulting in variability in care, uncertainty in decision-making, and inconsistent translation of evidence into practice.

Current literature provides limited guidance on how to integrate this complexity into stratified, individualised care pathways. Clinicians are required to navigate multiple, sometimes conflicting, intervention options with variable evidentiary support, increasing the risk of both under-treatment and low-value care. For families and caregivers, this uncertainty contributes to inconsistent advice and inequitable access to effective interventions.

### 1.3. Why a narrative review is the right format

The central challenge is no longer the absence of evidence, but its integration. The key questions, how interventions work, for whom, and under what conditions, require synthesis across clinical trials, and implementation research. A narrative review is therefore warranted to critically integrate evidence across domains, reconcile inconsistencies, and translate findings into clinically actionable guidance in real-world settings.

## 2. Objectives of the review

### 2.1. Primary aim

To synthesise the evolution of early intervention in CP and identify how advances in early detection, intervention design, and trial evidence inform a precision approach to care.

### 2.2 Secondary aims

We address the following questions:

1. How has early intervention in CP evolved across key domains, including *who* is treated, *when* intervention is delivered, *what* interventions are used, and *how* it works?
2. What is the current evidence from randomised controlled trials regarding the effectiveness of early, child-active interventions, and how consistent and trustworthy are these findings?
3. What patterns of treatment response emerge across trials, including differences by age, severity, motor subtype, and context?
4. How should current evidence inform a shift from a uniform approach to a precision early intervention model, focused on for *whom, when, how much*, and *where* interventions are most effective?
5. What are the key gaps in implementation, workforce capability, and caregiver support, and what directions should future research and service models prioritise?

## 3. Search strategy

We searched MEDLINE, Embase, CINAHL, and the Cochrane Library for literature published from January 2014 to March 2026. The search timeframe was selected to capture evidence generated following a summit held in Vienna 2014 that subsequently resulted in the introduction of the 2017 international early diagnosis guidelines. Search terms were structured using a PICOs framework. The Population included infants or children less than 2 years of age with “cerebral palsy” (or “high risk of cerebral palsy” or “high probability of cerebral palsy”). Intervention included “early intervention” or “early therapy” and all the named approaches specified in the early intervention guideline. Comparators or Outcomes were not restricted to allow capture of a broad range of clinically relevant endpoints, including motor, cognitive, and functional outcomes. Study designs were limited to randomised controlled trials and clinical practice guidelines. Reference lists of included articles were also hand searched to identify additional key studies. The findings are reported in accordance with the SANRA quality scale.^7^

## 4. The evidence base

### 4.1. Early intervention 1.0 (1970-2016)

Early intervention in CP was historically dominated by approaches such as Bobath, Neurodevelopmental Therapy (NDT), Neurofacilitation of Developmental Reaction (NFDR), and the Vojta method. A pivotal systematic review by Morgan and colleagues (2016)^8^ confirmed that, despite decades of widespread use, these approaches lacked evidence of efficacy for improving motor outcomes in infants with or at high risk of CP, and highlighted substantial methodological limitations in the evidence base. Collectively conceptualised here as “Early Intervention 1.0”, Bobath, NDT, NFDR, and Vojta approaches were characterised by ineffective choices of *what, when, how much, where,* and *who*. The *what* predominantly involved therapist-generated or reflexive movements not under volitional control. Bobath and NDT emphasised hands-on positioning and facilitation of “normal” movement while inhibiting “abnormal” patterns; NFDR focused on integrating abnormal reflexes to produce movement; and Vojta applied external pressure to elicit innate motor responses. Across approaches, infants were largely passive recipients rather than active agents generating and refining their own motor behaviour. The *when* was typically late, with intervention commencing after clear signs of motor delay, missing critical periods of heightened neuroplasticity. The *how much* was low intensity, typically delivered through regular clinic-based sessions without sufficient repetition to drive neuroplastic change. The *where* was predominantly clinic-based, with little opportunity for practice embedded in the child’s *everyday environments*, limiting generalisation and cumulative learning. The *who* was also problematic, with early studies frequently including heterogeneous samples of infants “at risk” of a range of developmental conditions, many of whom did not go on to have any disability or CP, resulting in low statistical power to detect true effects within the CP target population.

A notable exception was the curriculum-based trial by Palmer and colleagues known as “Learning Games”, which integrated motor and cognitive training and demonstrated superior outcomes compared to neurodevelopmental therapy in infants with the spastic diplegia sub-type.^9^ This study provided early signals that more effective intervention may require active training, and multi-domain learning delivered *earlier* with sufficient specificity for infants with CP. These insights marked the beginning of a shift towards a new conceptual model of early intervention, laying the foundation for contemporary approaches.

### 4.2. Early intervention 2.0 (2016-2021)

**Who and When:** Early Intervention 2.0 emerged in response to the limitations of Early Intervention 1.0, with a major focus on correcting the *who* by ensuring that the right infants were identified early and accurately so that intervention could be delivered during critical periods of neuroplasticity. This shift aligned with the principle of *earlier*, recognising that intervention delivered closer to the timing of brain injury harnesses heightened neuroplasticity, thereby making timing a key determinant of intervention effectiveness.

The development of the 2017 international clinical practice guideline for early diagnosis of CP established that diagnosis is both possible and recommended in infancy using a combination of standardized tools.^6^ The guideline synthesised high-quality evidence and concluded that CP or high risk of CP can be diagnosed accurately before 6 months’ corrected age using converging findings from neuroimaging, the General Movements Assessment (GMA), and the Hammersmith Infant Neurological Examination (HINE), each demonstrating strong predictive validity.^6^ This marked a paradigm shift from viewing infancy as a “silent period” to recognising it as a critical window for both detection and intervention.

Subsequent studies strengthened this evidence base. A large case–control study demonstrated that when results of these three assessments triangulate, diagnostic accuracy exceeds 97% sensitivity and 99% specificity, confirming that early diagnosis can be both reliable and clinically actionable.^10^ Each tool contributes complementary information, neuroimaging identifies structural brain injury, the HINE assesses neurological function, and GMA captures movement quality, supporting a triangulated detection approach.^10^

Following guideline publication, implementation efforts across multiple countries translated this evidence into practice. A systematic review of implementation studies found that guideline uptake significantly reduced the age of diagnosis, with a pooled reduction of approximately 7.5 months (95%CI -2.6, -12.4) and diagnosis commonly achieved before 12 months. In high-performing services, diagnosis can occur as early as 3–4 months, demonstrating what is achievable under optimal conditions.^11^

Collectively, Early Intervention 2.0 successfully addressed the *who and when* by enabling *earlier* and more accurate identification of infants with or at high risk of CP.

**What:** With the *who and when* defined, attention shifted to the *what*, developing interventions that align with principles of *engagement, exploration, and exercise*, rather than a passive receipt of movement therapy. A key methodological advance was possible because of the recruitment of more homogenous samples, enabling clearer interpretation of treatment effects.

At the same time, family-centred models of care began to emerge across socio-economic contexts. In LMICs, group-based early intervention programmes that focused on family and community support were gaining traction.^12,13^ In HIC contexts the COPCA (Coping with and Caring for Infants with Special Needs) programme^14^ was developed. These programmes represented an important conceptual shift from therapist-directed treatment towards coaching caregivers to support infant development within everyday activities and established caregiver coaching and family participation as important principles of contemporary early intervention.^12–14^ Phase II trials of COPCA did not demonstrate superior motor outcomes compared with traditional infant physiotherapy and the GRADE certainty of evidence remains very-low because of risk of bias, inconsistent findings, and indirectness, as study populations comprised infants at risk of a range of neurodevelopmental disabilities rather than confirmed CP.

A series of influential trials subsequently emerged,^15–20^ which we collectively term the “motor plus” studies because they extended intervention beyond the motor domain alone to target broader developmental domains. These studies reflected a growing recognition that, although CP is defined by a physical disability, it is a multidomain neurodevelopmental condition in which co-occurring impairments, including cognitive impairment, ADHD, autism, communication disorders, and vision impairments, often have as great an influence on long-term participation and quality of life as the motor disability itself. Consequently, these comorbidities became legitimate targets for early intervention rather than secondary consequences to be managed later.

For infants across all CP subtypes, including bilateral presentations, the Small Step program^16^ evaluated a structured, multi-domain intervention targeting mobility, hand use, and communication, delivered intensively in the home. While overall group effects were modest, infants receiving the intervention demonstrated more consistent developmental gains independent of severity, suggesting a potential protective effect for those most affected.

In parallel, the GAME (Goals–Activity–Motor Enrichment) approach^21,22^ operationalised principles of *experience, enriched environments*, and *everyday* practice, combining task-specific training, parent coaching, and environmental modification. Early pilot data demonstrated clinically meaningful improvements in motor outcomes, with subsequent trials confirming superior motor and cognitive outcomes at 12 months.^22^

For unilateral CP, Baby-CIMT^16^ demonstrated improved unimanual hand function through intensive, child-active practice. Similarly, multi-component upper limb interventions^19^ integrating motor and sensory training improved reach smoothness and somatosensory processing, reinforcing the importance of combining *exercise* with meaningful, multimodal *experiences*.

This body of work and other approaches to optimise infant learning was synthesised in the 2021 international clinical practice guideline for early intervention in CP,^23^ which defined best practice as intervention that is child-active, goal-directed, task-specific, and intensive, delivered within *enriched environments* and embedded in *everyday* routines with strong caregiver involvement. Passive, therapist-led approaches were explicitly not recommended.

Collectively, this evidence redefined what constitutes effective early intervention, shifting the field toward approaches that promote active *engagement, exploration*, and repeated practice within meaningful contexts.^24^

**How (Mechanism of Action)**: Across these studies, a consistent pattern emerged: effective interventions work by optimising the quality, quantity, and context of experience. This convergence culminated in a clear articulation of mechanisms, captured in the ‘E-words’ framework developed by Damiano^25^: *earlier, engagement, exploration, enriched environments, experiences, everyday*, and *exercise*.

Rather than discrete components, these E-words represent interacting mechanisms. *Engagement* and *exploration* drive active learning; *enriched environments* and *everyday* contexts ensure sufficient opportunities for practice; and *exercis*e and repetition provide the dose required for neuroplastic change. Together, they operationalise *experience-dependent plasticity*, where repeated, self-initiated activity strengthens neural pathways. These principles position early intervention as a process of optimising *experience* to drive adaptive brain and behavioural change, rather than attempting to correct impairments or normalise movement and function.

**Synopsis:** Across Early Intervention 2.0 (2016–2021), the strongest signal was a shift toward child-active, context-based, parent-implemented, and task-specific interventions. The most consistent early intervention benefits were observed in trials of GAME,^21–23^ Baby-CIMT,^16^ and multicomponent upper-limb interventions.^20^ In contrast, the Small Step trial^18^ demonstrated subgroup effects, suggesting greater benefit for infants with more severe impairment, while the COPCA LEARN2MOVE trial^14^ showed no clear superiority over NDT comparison care.

### 4.3. Early intervention 3.0 (2021-2026)

Early Intervention 3.0 is characterised by the emergence of large, pragmatic randomised controlled trials^25–30^ evaluating early intervention in infants with or at high risk of CP across diverse health systems, including both high-income and low- and middle-income settings. These trials represent a progression from Early Intervention 2.0 efficacy studies to real-world evaluation of early, CP-specific interventions delivered at scale.

**Who and When**: Recent diagnostic studies confirm that early detection is feasible beyond high-income settings. Prospective cohort studies in Bangladesh^31^ and India^32^ demonstrate that accurate identification of CP is possible as early as 3 months using recommended tools, even in resource-constrained environments, with strong predictive validity and scalability. These findings indicate that early diagnosis can be achieved across diverse health systems. Across intervention trials, this has enabled recruitment of infants within the first year of life, including those with confirmed CP and clearly defined high-risk profiles.

Early intervention programs also play a complementary role in supporting diagnostic pathways when combined with close clinical surveillance. In many health systems, diagnostic services and early intervention are delivered in parallel rather than within the same setting. However, regular monitoring of infants receiving early intervention, through repeated developmental assessments and clinical review, can support earlier recognition of atypical trajectories and facilitate timely referral for diagnostic evaluation. This reduces uncertainty for families, enables more accurate prognostic counselling, and supports earlier access to specialised intervention trials and services.

Close surveillance alongside early intervention also enables earlier identification of infants with conditions that may mimic CP. Disorders such as hereditary spastic paraplegia, leukodystrophies, mitochondrial disease, and congenital myopathies may initially present with similar motor features. Coordinated pathways linking early intervention with medical and diagnostic services, including clinical assessment, developmental testing, neuroimaging where appropriate, and targeted genetic or metabolic investigations, can support earlier differentiation than routine care alone.

The implications of earlier and more precise differentiation are substantial. For some genetic conditions, disease-specific treatments are now available or emerging. Even when curative treatments are not available, establishing an accurate diagnosis informs surveillance for associated complications, guides genetic counselling, and enables access to condition-specific support networks. At a systems level, earlier diagnostic clarification may improve resource allocation, while at the family level it may support caregiver confidence and more responsive parent–infant interactions.

**What:** Within this context, interventions were predominantly child-active, task-specific, and caregiver-implemented, typically delivered at moderate to high dose and embedded within everyday environments. Across pragmatic trials, overall between-group differences were frequently not statistically significant on primary outcomes, with improvements observed in both intervention and comparison groups. However, predefined subgroup analyses demonstrated statistically significant effects within specific strata, including differences by age at intervention commencement, motor severity, and socioeconomic context.

It is very important to acknowledge that comparator conditions in 3.0 commonly involved active, motor learning–based interventions, reflecting changes in usual care compared with Early Intervention 2.0 studies. As a result, many trials in 3.0 compared different active approaches head-to-head, rather than active versus passive interventions.

### 4.4. Trustworthiness of the evidence

The certainty of evidence ranged from low to moderate (GRADE).^33^ Moderate-certainty evidence supports improvements in upper limb function, particularly in unilateral CP, where multiple trials demonstrate gains in hand use following child-active, task-specific interventions such as CIMT and bimanual training.^26^ These evidence-based approaches now need to be implemented within usual care.

In contrast, evidence for gross motor and cognitive outcomes was less consistent across trials. Certainty was downgraded because of risk of bias inherent to behavioural interventions, imprecision in early-phase and subgroup analyses, and inconsistency across heterogeneous populations, interventions, and contexts. However, some of the apparent inconsistency in cognitive outcomes may also reflect limitations of outcome measurement. Measuring cognition in infants with CP is challenging because many of the most used assessments, including the Bayley Scales of Infant and Toddler Development, have recognised limitations in children with motor impairment.^34^ Successful completion of several items on the cognitive subscale depends on motor performance, meaning cognitive scores may underestimate true cognitive ability rather than true treatment effects.^34^

Risk of bias: Risk of bias (RoB 2)^35^ across trials was generally rated as “some concerns”. The most common source of bias arose from deviations from intended intervention, reflecting the inability to blind participants and therapists in caregiver-mediated interventions. Additional sources included missing outcome data and lack of pre-specified analysis plans.

More recent trials demonstrated improved methodological rigour compared with Early Intervention 2.0 studies, including: concealed allocation; blinded outcome assessment; use of objective outcome measures; and clearer inclusion criteria case definitions for CP and high-risk populations.

To support transparency and enable comparison across studies, key characteristics, outcomes, and methodological quality of trials in Early Intervention 2.0 and 3.0 are summarised in Tables 1 and 2.

**Table 1.** Randomised controlled trials in Early Intervention 2.0 and 3.0.

Citation	Design	Participants	Location	Results and interpretation	E-words	GRADE	RoB 2
**EARLY INTERVENTION 2.0 (2016-2021)**							
Chamudot 2018 (CIMT v BIM)^15^	RCT n=33	CP Unilateral; n=17 mCIMT vs n=16 BIM	HIC Israel	**Outcome:** No significant between-group difference; hand function improved equally from mCIMT or BIM, on the Mini-AHA	Earlier engagementexperiences everyday	Low	Some concerns
				**Dose:** ∼ 47 hours			
				**Comparison:** Motor learning vs motor learning			
				**Interpretation:** mCIMT and BIM appear equally effective, consistent with findings in older children			
Eliasson 2018 (Baby-CIMT)^16^	RCT n=37	CP Unilateral; n=18 Baby-CIMT vs n=13 massage	HIC Sweden	**Outcome:** Baby-CIMT conferred superior unimanual hand function improvements (p=0.041), on the HAI. Baby-CIMT benefits persisted at 18 months, on the AHA (51 vs 24; no CI reported)	Earlier engagementexperiences	Low	Some concerns
				**Dose:** ∼36 hours			
				**Comparison:** Motor learning vs non-motor learning			
				**Interpretation:** Baby-CIMT produced superior results to passive movement			
Harbourne 2021 (START-Play)^17^	RCT n=112	ND incl CP; n=57 START-PLAY vs n=55 UC	HIC USA	**Outcome:** Significant subgroup effects were observed. START-PLAY conferred superior improvements in cognition, fine motor, and reaching in infants with the most severe delays. START-PLAY benefits persisted at 12 months. START-PLAY conferred superior improvements in communication in milder children but not motor	Engagementexploration experiences enriched environment	Low	Some concerns
				**Dose:** ∼18 hours			
				**Comparison:** Motor learning vs non-motor learning			
				**Interpretation:** Task-specific practice appears to benefit those with the most severe motor impairments in HICs			
Hielkema 2020 (LEARN 2MOVE)^14^	RCT n=43	High risk CP; n=23 COPCA vs n=20 NDT	HIC Nether-lands	**Outcome:** No between-group difference (p>0.05), both groups improved equally in motor, cognition, and behaviour, on the IMP.	Engagementeveryday experiences	Low	Some concerns
				**Dose:** ∼39 hours			
				**Comparison:** Coaching vs non-motor learning			
				**Interpretation:** COPCA Coaching used the E-word principles, but potentially was not task-specific enough in focus to produce a result			
Holmström 2019 (SMALL STEP)^18^	RCT n=39	High risk CP or ND; n=19 Small Step vs n=20 UC	HIC Sweden	**Outcome:** No overall effect; significant subgroup effects were observed. SMALL-STEP conferred superior improvements in in infants with the most severe delays (p=0.02); SMALL-STEP benefits persisted at 2 years.	Exploration enriched environment everyday experiences	Low	Some concerns
				**Dose:** ∼28 hours			
				**Comparison:** Motor learning vs motor learning			
				**Interpretation:** Task-specific practice appears to benefit those with the most severe motor impairments in HICs			
Hwang 2020 (mCIMT)^19^	RCT n=24	CP Unilateral; n=12 mCIMT vs n=12 no therapy	HIC Korea	**Outcome:** mCIMT conferred superior hand function improvements in how often (p=0.048) on the PMAL, how well (p=0.008) on the PDMS-2 (p=0.014); and how much on accelerometry	Exercise everyday engagement	Low	Some concerns
				**Dose:** ∼30 hours			
				**Comparison:** Motor learning vs nothing			
				**Interpretation:** mCIMT is effective			
Maitre 2020 (multi-component training)^20^	RCT n=73	CP Unilateral or bilateral asymmetric; n=37 multi-component training (CIMT + BIM + other) vs n=36 bimanual play	HIC USA	**Outcome:** Multi-component training conferred superior reach smoothness (p<0.001); fine motor (p=0.004); and somatosensory (p=0.04, unadjusted)	Exercise, engagement experiences enriched environment	Moderate	Low
				**Dose:** ∼182 hours			
				**Comparison:** high dose motor learning vs low dose motor learning			
				**Interpretation:** Multi-component training is effective and feasible			
Morgan 2015 (GAME)^21^	Pilot RCT n=13	High risk CP; n=6 GAME vs n=7 UC	HIC Australia	**Outcome:** GAME conferred superior motor skills on the PDMS-2 TMQ MD=8.05 (95% CI 3.88–12.23; p<0.001)	Earlier engagementeveryday enriched environment	Low	High
				**Dose:** ∼140 hours			
				**Comparison:** Motor learning vs non-motor learning			
				**Interpretation:** GAME appears effective			
Morgan 2016 (GAME)^22^	RCT n=30	CP or High risk CP; n=15 GAME vs n=15 UC	HIC Australia	**Outcome:** GAME conferred superior motor skills on the PDMS-2 raw (p=0.01); TMQ (p=0.05) and GMFM (p=0.05); and superior cognitive skills on the BSID-3 (p=0.03)	Earlier engagementeveryday enriched environment	Low	Some concerns
				**Dose:** ∼216 hours			
				**Comparison:** Motor learning vs non-motor learning			
				**Interpretation:** GAME appears effective			
**EARLY INTERVENTION 3.0 (2021-2026)**							
Benfer 2024 (LEAP-CP)^25^	RCT n=153	High risk CP; n=60 LEAP vs n=58 health advice	LMIC India/Bangladesh	**Outcome:** No overall effect; significant subgroup effects were observed. LEAP conferred superior motor skills on the PEDI-CAT MD=4.0 (95% CI 1.4–6.5; p=0.003) in infants with the mildest CP (GMFCS I-II)	Engagementeveryday experiences enriched environment	Moderate	Some concerns
				**Dose:** ∼15 hours			
				**Comparison:** Motor learning vs nothing			
				**Interpretation:** LEAP appears more effective in children with milder physical disability. In LMIC settings, where severe disability is more prevalent and associated with a higher burden of comorbidities, responsiveness to intervention may be reduced. Additionally, the intervention may have lacked sufficient specificity when delivered by community disability workers.			
Boyd 2025 (REACH)^26^	RCT n=96	Unilateral CP; n=46 Baby-CIMT vs n=50 Baby-BIM	HIC Australia/USA	**Outcome:** No significant between-group difference on the HAI; hand function improved equally from mCIMT or BIM. Significant subgroup effects were observed in those who started treatment earlier.	Earlier engagement experiences everyday	Moderate	Some concerns
				**Dose:** ∼75 hours			
				**Comparison:** Motor learning vs motor learning			
				**Interpretation:** mCIMT and BIM appear equally effective, consistent with findings in older children			
Carton de Tournai 2024 (baby HABIT-ILE)^27^	RCT	Unilateral CP; n=24 baby HABIT-ILE vs n=24 NDT	HIC Belgium	**Outcome:** baby HABIT-ILE conferred superior hand use skills on the Mini-AHA (group×time p=0.008); and goal achievement on the COPM (p<0.001); but no significant differences in gross motor function on the GMFM	Exercise engagementexploration enriched environment	Moderate	Some concerns
				**Dose:** ∼50 hours			
				**Comparison:** Motor learning vs non-motor learning			
				**Interpretation:** baby HABIT-ILE appears effective for improving hand function but not gross motor, consistent with HABIT-ILE findings in older children (Sakzewski)			
Lucas 2024 (Best Start)^28^	RCT	High risk CP/ND; n=15 Best Start vs n=15 UC positioning	HIC Australia	**Outcome:** No significant between-group difference on the AIMS: MD=-0.2 (95% CI -2.4 to 2.0); parent-reported benefit favoured Best Start therapy	Earlier everyday experiences	Low	Some concerns
				**Dose:** ∼100 hours			
				**Comparison:** Motor learning vs non-motor learning			
				**Interpretation:** Best Start appears feasible and acceptable. Refinement of inclusion criteria is advised for future studies			
Morgan 2024 (GAME conference abstract)^29^	RCT n=302	CP or High Risk of CP; n=150 GAME vs n=152 UC	HIC Australia	**Outcome:** No significant between-group difference on PDMS-2. Significant subgroup effects were observed. GAME conferred superior function on the PEDICAT in those with CP and severe physical disability (GMFCS III–V), and superior adaptive behaviour in those from low socioeconomic (SES) status	Earlier engagement enriched environment everyday	Moderate	Low
				**Dose:** ∼100+ hours			
				**Comparison:** Motor learning vs motor learning			
				**Interpretation:** Interpretation is influenced by intercurrent events, including COVID-19–related disruptions to intervention delivery and a shift in UC from non–motor learning approaches in the 2015-2016 pilot studies to motor learning–based care in the GAME trial. GAME appeared to confer the greatest benefit in the most vulnerable subgroups, those with severe physical disability and low socioeconomic status, consistent with subgroup findings from Small Step and START-Play.			
Prosser 2024 (iMOVE)^30^	RCT n=42	CP; n=20 iMOVE vs n=21 UC	HIC USA	**Outcome:** No significant between-group difference on GMFM-66; both groups improved equally	Exercise engagement exploration	Moderate	Some concerns
				**Dose:** ∼54 hours			
				**Comparison:** Motor learning vs non-motor learning			
				**Interpretation:** The limited transfer of learning from clinic-based use of the iMOVE device to home-based practice, unlike other motor learning interventions, may reduce its effectiveness

**Table 2.** GRADE summary of findings.

Outcome	No. of studies (participants)	Effect (summary of findings)	Certainty of evidence (GRADE)	Reasons for downgrading
Upper limb function (unilateral CP)	6 RCTs (n≈260)	Consistent improvements in hand function with child-active interventions (Baby-CIMT, mCIMT, BIM, HABIT-ILE, multi-component training). No superiority between active approaches (e.g., CIMT vs BIM), but superiority over passive/no therapy comparators.	⨁⨁⨁◯ Moderate	Risk of bias (lack of blinding), imprecision (small sample sizes), but consistent direction of effect
Gross motor outcomes (PDMS-2, GMFM)	9 RCTs (n≈500)	Early trials (GAME, Multi-component) show significant improvements. Later pragmatic trials (LEAP, REACH, iMOVE, Best Start) show no overall between-group differences, with subgroup effects observed.	⨁⨁◯◯ Low–Moderate	Inconsistency (positive vs null trials), imprecision (small early trials), heterogeneity (population, comparator, context)
Cognitive outcomes	3 RCTs (n≈150)	Improvements reported in GAME and START-Play (particularly in severe subgroups); inconsistent across trials and populations.	⨁⨁◯◯ Low	Indirectness (mixed high-risk populations), inconsistency, small sample sizes
Real-world function/participation (PMAL, PEDI-CAT, COPM)	6 RCTs (n≈350)	Improvements in real-world use and goal attainment (PMAL, COPM), but population-level effects inconsistent; subgroup effects common (e.g., LEAP, GAME, START-Play).	⨁⨁◯◯ Low–Moderate	Inconsistency (subgroup-driven effects), measurement variability, some risk of bias
Effect of dose and intervention intensity	Across all RCTs	Higher-dose interventions in EI 2.0 associated with improved outcomes; EI 3.0 trials show no consistent superiority between high-dose active interventions, suggesting threshold effects.	⨁⨁◯◯ Low	Indirectness (dose not primary outcome), inconsistency across trials
Safety and feasibility	6+ RCTs (n≈400)	Home-based, parent delivered interventions generally safe and feasible; high adherence reported in structured programs (e.g., GAME, HABIT-ILE, Multi-component).	⨁⨁⨁◯ Moderate	Some reporting variability

## 5. Shifts in early intervention over time (1.0 → 3.0)

**Figure 1.**
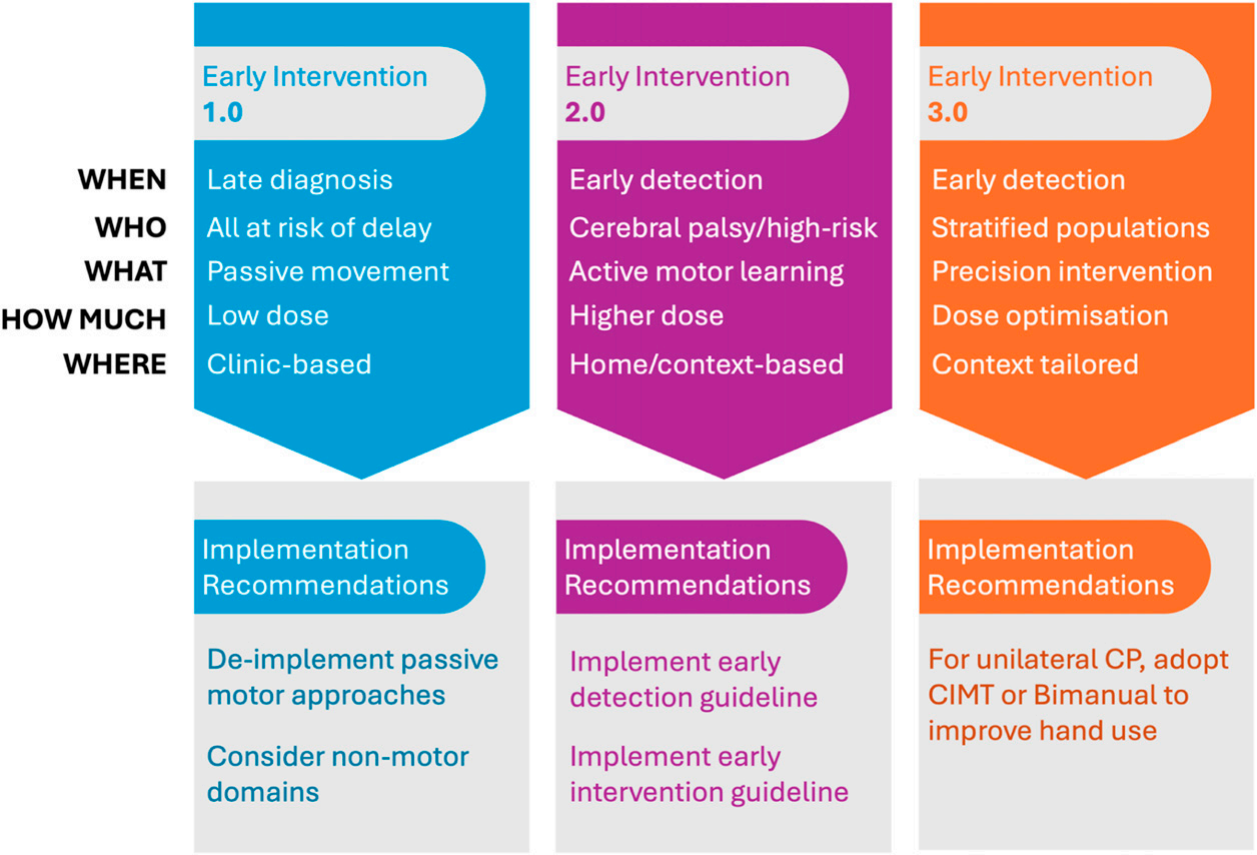
Conceptual and temporal shifts in cerebral palsy early intervention.

Across the evolution from Early Intervention 1.0 to 3.0, the field has undergone a fundamental transition, from passive, late, and low-dose approaches to early, active, and increasingly contextually embedded interventions. However, the most important recent shift is not simply in intervention content, but in how treatment effects are understood and interpreted (Figure 1).

Early Intervention 2.0 established that *early*, child-active, and task-specific interventions can improve outcomes when delivered during periods of heightened neuroplasticity. These findings were largely derived from small, controlled trials, where active interventions were compared with passive or low-intensity care, and consistent between-group differences were observed. In contrast, Early Intervention 3.0 has introduced larger, pragmatic trials conducted across diverse health systems, including LMICs, with more heterogeneous populations and more active comparator conditions. Within this context, overall between-group differences are frequently attenuated or absent.

This apparent reduction in effect does not indicate a loss of efficacy. Rather, it reflects three interrelated shifts in the evidence base. First, usual care has evolved in locations detecting CP early and conducting early intervention clinical trials. Comparator groups now commonly receive active, motor learning–based interventions, reducing the contrast between experimental and control conditions. Second, large CP study populations are being recruited and consistent with the definition are heterogeneous, encompassing a broad spectrum of severity, comorbidity, and environmental context, which dilutes average treatment effects. Third, interventions are delivered in real-world settings, where implementation variability, caregiver capacity, and contextual constraints influence outcomes.

A defining feature of Early Intervention 3.0 is a shift from population-level treatment effects to an emerging signal of subgroup-specific treatment responses. Across trials, benefits are consistently observed within specific strata rather than across entire study populations. These strata include *earlier* age at intervention commencement, severity of motor impairment, topography of motor impairment, and socioeconomic or environmental context. For example, in high-income settings, infants with more severe physical disability appear to benefit most from intensive, task-specific, and enriched interventions, whereas in LMIC settings, infants with milder physical disability (GMFCS I-II) demonstrate greater responsiveness,^25^ likely reflecting differences in baseline opportunity for experience and stimulation. These patterns indicate that treatment response is not uniform, but contingent on the interaction between the individual, the intervention, and the environment.

In parallel, the role of dose has become more nuanced. While early studies suggested a linear dose–response relationship, where more is better, more recent trials indicate that dose alone does not explain outcomes. High-dose interventions appear superior when compared to non-motor learning therapies, but do not consistently outperform lower-dose active motor learning comparators. This suggests the presence of threshold effects, a concept supported by evidence in older children with CP,^36,37^ and underscores the importance of intervention specificity, timing, and contextual embedding of practice. Interventions that are integrated into *everyday* routines and supported by caregiver coaching appear more likely to achieve sufficient repetition and generalisation, whereas clinic-based or device-dependent approaches may show limited transfer to real-world function.

The increasing inclusion of LMIC settings has further highlighted the importance of context. Intervention effects vary according to health system capacity, workforce expertise, and baseline environmental enrichment. These findings suggest that early intervention is not a fixed treatment package, but a context-sensitive process that interacts with available system resources, parental resources, and opportunities for learning.

Another notable shift is in outcome measurement. Trials in 1.0 focused primarily on impairment and capacity-based measures, whereas more recent studies increasingly assess real-world function, and goal attainment. This has revealed a gap between improvements in motor capacity and translation into *everyday* performance, identifying generalisation of learning as a key challenge for intervention design.

Taken together, these findings indicate that early intervention for CP is effective, but not universally so. The transition from Early Intervention 2.0 to 3.0 reflects a shift from an efficacy paradigm, focused on whether interventions work, to a precision paradigm, focused on for whom, when, how much, and where interventions are most effective. In this model, variability in treatment response is not viewed as noise, but as a critical signal that can be used to optimise and individualise care.

## 6. Future directions

The next phase of the field must move beyond demonstrating efficacy toward optimising timing, targeting, delivery, and implementation at scale.

### 6.1. Precision early intervention: Timing and stratified trial design

Future clinical trials are now positioned to recruit large cohorts of infants with CP within the first months of life, enabled by advances in early diagnosis. This creates a unique opportunity to rigorously test the timing of intervention during critical periods of neuroplasticity. Trials such as the Very Early Intervention Program (VIP) (ACTRN12624001417572p); the GO-PLAY trial (Goal Oriented ParentaL supported home ActivitY (NCT05883969); and Daily and Weekly Rehabilitation Delivery for Young Children With Gross Motor Delays (DRIVE) (NCT02857933) are beginning to address these questions, but further work is required to define optimal windows for intervention initiation.

In parallel, emerging trials are also advancing the design of early intervention itself. For example, the CONTRACT trial (NCT04250454) is evaluating a multicomponent, caregiver-mediated approach, reflecting a complementary focus on how interventions should be configured, alongside when and where they should be delivered.

A key methodological priority is the shift from treating CP as a single heterogeneous condition to explicitly stratifying participants into biologically and clinically meaningful subpopulations. Evidence from Early Intervention 3.0 demonstrates that treatment effects are frequently confined to subgroups defined by age, severity, motor subtype, or contextual factors. Future trials should therefore be designed a priori to test differential responses across strata, using adaptive and precision trial methodologies. Without this shift, true treatment effects risk being diluted when analysed across heterogeneous populations.

### 6.2. Embedding caregiver mental health and parenting supports early

A critical and currently unmet priority is the inclusion of structured, evidence-based caregiver support at or near the time of diagnosis. Caregiver mental health is a key determinant of child outcomes yet remains largely unaddressed in current models of care. Caregivers of children with CP experience mental health challenges at approximately twice the population rate, and one in three children with CP develops their own mental health difficulties.^36^ Despite this, funding models are directed almost exclusively toward the child, with no dedicated support for caregivers. Although family-oriented models of care and parent coaching have been explored in both high-income and low- and middle-income settings, the evidence remains limited and no approach has yet been established as standard care.^12–14^

The shift to early diagnosis, now occurring before 6 months of age in many settings, creates a critical window for preventative intervention. During this period, both neuroplasticity and caregiver–infant relational dynamics are highly responsive to intervention. Decades of research demonstrate that parental engagement improves caregiver self-efficacy (effect size 0.61; 95% CI 0.59–0.63)^38^ and child developmental outcomes (0.24; 95% CI 0.20–0.29),^39^ yet current early intervention models do not systematically incorporate the caregiver–infant dyad.

Existing parenting interventions in CP are typically delivered later in childhood, after behavioural challenges have emerged, and are inconsistently implemented. In contrast, evidence from adjacent populations demonstrates the potential impact of early caregiver support. One parent-empowerment program is the Parenting Acceptance and Commitment Therapy (PACT) program, currently being evaluated in a clinical trial.^38^ This is designed for parents or other caregivers of children with newly diagnosed neurodisabilities. The goal of the program is to help parents feel more competent and empowered to handle the many challenges that arise.

Emerging work is also beginning to examine the role of caregiver–infant dyadic reciprocity as a potential mechanism underpinning early intervention. Ongoing observational studies^39^ are investigating how responsive, reciprocal interactions between caregivers and infants may influence developmental trajectories, but results are not yet available.^40,41^

A landmark Australian trial in preterm infants showed that an 8-week early intervention focused on parental wellbeing and responsive caregiving produced sustained improvements in caregiver mental health and child behaviour up to 13 years later.^42^ To date, no CP trials have tested or costed structured caregiver support delivered at the time of diagnosis. Addressing this gap represents a major opportunity to improve both child and family outcomes, while supporting workforce participation and reducing long-term reliance on welfare systems.

### 6.3. Accelerating discovery through global collaboration and innovative designs

The increasing complexity of early intervention research necessitates new approaches to trial design and delivery. The Cerebral Palsy Global Clinical Trials Network (https://cerebralpalsy.org.au/our-research/cp-global-clinical-trials-network/) provides a coordinated international platform to accelerate trial conduct, increase sample sizes, and enhance generalisability across diverse health systems.^43,44^ By harmonising protocols, outcome measures, and data infrastructure, the network enables multi-site, adaptive, and platform trials capable of testing multiple interventions and subgroups simultaneously.

Such approaches are particularly important in early intervention, where rapid developmental change, heterogeneity, and contextual variability challenge traditional trial designs. Leveraging global collaboration will be essential to generate sufficiently powered evidence, reduce duplication, and accelerate translation of findings into clinical practice.

Complementary infrastructure is also emerging to support these efforts. Population-based initiatives such as the Global LMIC Cerebral Palsy Register (https://www.glmcpr.org) provide critical datasets for case identification, longitudinal follow-up, and inclusion of underrepresented populations. In parallel, collaborative networks such as CP360 (https://cp360.org) facilitate global coordination and knowledge exchange across diverse settings. Together, these initiatives strengthen the infrastructure required for efficient recruitment, stratification, and implementation of large-scale early intervention trials.

### 6.4. Closing the allied health workforce implementation gap

A persistent barrier to the impact of early intervention is the gap between evidence and implementation. Advances in Early Intervention 3.0 have outpaced workforce capability, with many allied health professionals lacking the training, confidence, and resources to deliver contemporary, child-active, and contextually embedded interventions.

Addressing this gap requires not only workforce development, but a broader shift in models of care. Early Intervention 3.0 necessitates a transition from clinician-delivered services toward system-enabled models, in which intervention is distributed across caregivers, communities, and digital platforms. In this model, clinicians act not only as providers of therapy, but as facilitators of learning, coaches for caregivers, and coordinators within multidisciplinary systems.

Operationalising this shift requires coordinated action across three domains: (1) strengthening workforce capability through scalable training and decision-support; (2) adopting flexible, hybrid delivery models that extend intervention beyond traditional service settings; and (3) aligning funding, policy, and care pathways to support early, intensive, and family-centred care.

Scalable knowledge translation solutions are central to this approach. Digital eLearning platforms such as CPAdvance™ (https://cpadvance.org) provide a mechanism to translate evidence into practice by delivering structured guidance, decision support, and training aligned with current best evidence. Such tools can support consistent, high-quality implementation across diverse settings, including those with limited specialist expertise.

Without targeted investment in workforce development, system redesign, and implementation infrastructure, the benefits of Early Intervention 3.0 will remain unevenly distributed. Bridging this gap is therefore essential to ensure that advances in early detection and intervention translate into improved outcomes for all children and families.

#### 6.5. Limitations

This review has several limitations. As a narrative review, it does not follow a fully systematic methodology, and therefore may be subject to selection bias, incomplete retrieval of studies, and lack of reproducibility compared with systematic reviews. Although we used structured search strategies and explicit inclusion criteria, formal quantitative synthesis was not undertaken. The evidence base itself is heterogeneous, with variation in populations, interventions, dose, and outcome measures, which limits direct comparability across trials. In addition, many early intervention studies are small, include mixed “high-risk” populations rather than confirmed CP, and are subject to methodological challenges inherent to behavioural interventions, including lack of blinding and variability in implementation. Finally, emerging evidence from recent large trials is still evolving, with some findings available only in abstracts or ongoing studies, which may influence conclusions as the field develops further.

## 7. Conclusion

The future of early intervention in CP lies not in discovering new interventions alone, but in delivering the right intervention, to the right child, at the right time, and within the right context, supported by caregivers, enabled by systems, and implemented at scale.

## Data Availability

No datasets were generated or analysed during the current study.*
